# Short- and long-term effects of concurrent aerobic and resistance training on circulating irisin levels in overweight or obese individuals: a systematic review and meta-analysis of randomized controlled trials

**DOI:** 10.7717/peerj.17958

**Published:** 2024-09-19

**Authors:** Yang Cheng, Jing Ma, Shumin Bo

**Affiliations:** Capital University of Physical Education And Sports, Beijing, Haidian, China

**Keywords:** Concurrent training, Aerobic training, Resistance training, Overweight, Obesity, Irisin

## Abstract

**Background:**

Concurrent training (CT) is emerging as a practical and effective approach to enhance body composition, cardiovascular function, and muscle mass, thereby elevating overall individual health. This study aims to systematically investigate the effects of short- and long-term concurrent aerobic and resistance training on circulating irisin levels in overweight or obese individuals.

**Methodology:**

The electronic databases, including China National Knowledge Infrastructure, PubMed, Embase, Wan Fang Database, and Web of Science, were systematically searched for articles on “concurrent training” and “irisin” published from their inception to 30 November 2023. The pooled effect size was determined using standardized mean difference (SMD) and corresponding 95% confidence intervals (CIs). The study protocol received registration with the International Prospective Register of Systematic Reviews (CRD42023494163).

**Results:**

All nine studies, encompassing a total of 264 participants, were randomized controlled trials and met the eligibility criteria. Results indicate that short- and long-term concurrent training moderately increased circulating irisin levels compared to the control group (SMD = 0.56, 95% CI [0.33–0.80], *p* = 0.00; *I*^2^ = 36.6%, heterogeneity *p* = 0.106). Subgroup analyses revealed that both equal to or less than 10 weeks (SMD = 0.78, 95% CI [0.18–1.37], *p* = 0.01; *I*^2^ = 62.3%, heterogeneity *p* = 0.03) and more than 10 weeks (SMD = 0.45, 95% CI [0.14–0.76], *p* = 0.00; *I*^2^ = 0%, heterogeneity *p* = 0.54) of concurrent training significantly increased circulating irisin levels in overweight or obese individuals. There were no significant between-group differences (*I*^2^ = 0%, *p* = 0.34). Additionally, concurrent training significantly increased irisin levels in overweight or obese participants (SMD = 1.06, 95% CI [0.34–1.78], *p* = 0.00; *I*^2^ = 50.6%, heterogeneity *p* = 0.13) and in type 2 diabetes patients (SMD = 0.70, 95% CI [0.30–1.10], *p* = 0.00; *I*^2^ = 0%, heterogeneity *p* = 0.99). However, no significant effect was observed in patients with metabolic syndrome (SMD = 0.21, 95% CI [−0.25–0.68], *p* = 0.37; *I*^2^ = 38.7%, heterogeneity *p* = 0.18). There were significant between-group differences (*I*^2^ = 53.9%, *p* = 0.11). Lastly, concurrent training significantly increased circulating irisin levels in overweight or obese individuals aged 45-60 years (SMD = 0.56, 95% CI [0.25–0.86], *p* = 0.00; *I*^2^ = 6.5%, heterogeneity *p* = 0.38), and a significant increase in irisin levels was observed 12 h post-intervention (SMD = 0.70, 95% CI [0.35–1.05], *p* = 0.00; *I*^2^ = 0%, heterogeneity *p* = 0.74). However, none of the above categorical variables showed significant between-group differences.

**Conclusions:**

Short- and long-term concurrent training can effectively improve circulating irisin levels in overweight or obese individuals. However, the effects of short- and long-term concurrent training should consider the participants’ health status, age, and the timing of post-exercise measurements to maximize health benefits.

## Introduction

A recent epidemiological study on obesity has shown that the global prevalence of overweight and obesity has surged by more than 50% (Chooi, Ding & Magkos, 2019). This alarming trend is primarily driven by heightened energy intake coupled with a sedentary lifestyle, which leads to increased body weight and further complicates various health issues. These issues include not only obesity itself but also metabolic syndrome (MetS) (Huh et al., 2014), cardiovascular disease (Arhire, Mihalache & Covasa, 2019), and type 2 diabetes mellitus (T2DM) (Rad et al., 2023). As a result, this situation emerges as a significant and concerning public health issue (Arhire, Mihalache & Covasa, 2019). Within the framework of muscle function regulation, myokines synthesized by muscle cells and released during muscle contraction may play a key role in improving this condition (Lee & Jun, 2019). The myokines identified to date encompass peptides, growth factors, and small organic acids (Das, Graham & Cardozo, 2020), such as irisin, interleukins, brain-derived neurotrophic factor (BDNF), β-aminoisobutyric acid (BAIBA), and myostatin (MSTN) (Gonzalez-Gil & Elizondo-Montemayor, 2020). These myokines facilitate communication between skeletal muscles and other organs, including the skeleton, liver, adipose tissues, pancreas, and nervous system (Huh, 2018). These interactions can elicit favorable physiological and metabolic effects, contributing to the establishment of a positive metabolic state and the optimization of overall energy metabolism (Gonzalez-Gil & Elizondo-Montemayor, 2020).

Irisin, named after the Greek goddess Iris, is regulated by the complex modulation of peroxisome proliferator-activated receptor gamma coactivator 1-alpha (PGC-1α). PGC-1α, influenced by exercise, can regulate the release of irisin through the modulation of the membrane protein fibronectin type III domain-containing protein 5 (FNDC5). FNDC5 has a complex structure, including an N-terminal sequence, an irisin domain, a transmembrane region, and a cytoplasmic C-terminal domain. Irisin, a 112-amino acid peptide cleaved and secreted from FNDC5, primarily forms a continuous intersubunit β-sheet dimer. This peptide is proteolytically cleaved from FNDC5 in skeletal muscle cells and then enters the bloodstream to exert its physiological effects (Boström et al., 2012; Liu et al., 2024). The secretion of irisin predominantly occurs in skeletal muscle cells (Roca-Rivada et al., 2013; Yu et al., 2019). Its primary role is to induce browning of white adipose tissue through the activation of mitochondrial uncoupling protein 1 (UCP1) (Grygiel-Górniak & Puszczewicz, 2017), thereby increasing energy expenditure and heat dissipation (Bonfante et al., 2017a). Studies have shown that elevated FNDC5/irisin levels in middle-aged obese men contribute to improve lipid metabolism and decelerate T2DM progression (Bonfante et al., 2017a; Bonfante et al., 2017b). Additionally, the elevation of irisin is observed in individuals exhibiting MetS features (Arhire, Mihalache & Covasa, 2019). While many findings regarding the role of irisin are association-based, these studies have highlighted the crucial function of irisin, a vital myokine, in inhibiting fat accumulation, modulating energy metabolism, and enhancing glucose regulation (Duan et al., 2016). Since PGC-1α and FNDC5, two key irisin precursors, are primarily expressed in skeletal muscle, circulating irisin levels are closely linked to exercise training (Huh et al., 2014).

Kim et al. (2016) observed that after an 8-week training program involving 28 overweight/obese individuals, the irisin levels significantly increased in the aerobic training (AT) group, whereas no significant change was observed in the resistance training (RT) group. Jandova et al. (2021) and Mohammad Rahimi, Hejazi & Hofmeister (2022) further support the positive impact of exercise on irisin levels. Recent systematic review and meta-analysis studies by Torabi et al. (2024) have shown that compared to control groups, high-intensity interval training (HIIT) significantly increases circulating irisin levels in overweight or obese individuals.

Intriguingly, incorporating RT and AT simultaneously, also known as combined training or concurrent training (CT), is emerging as a pragmatic and efficacious approach for improving body composition, cardiorespiratory fitness, muscle mass, aerobic capacity, and consequently overall fitness levels in individuals (Amaro-Gahete et al., 2019; Bao et al., 2020; Bouamra et al., 2022; Hendrickse et al., 2021; Müller et al., 2021). To our knowledge, although previous research has demonstrated the impact of physical exercise on irisin levels, there is currently no meta-analysis specifically focusing on the effects of CT on overweight or obese individuals, and whether CT exerts interference effects (potential impact of AT on RT adaptation after more than 10 weeks of CT) on them remains unclear. This is particularly important for overweight or obese individuals as it may contribute to improving their health status. In light of these considerations, our study aimed to utilize a meta-analysis approach to investigate the impacts of short- and long-term CT on circulating irisin levels in overweight or obese individuals.

## Survey Methodology

This systematic review and meta-analysis followed the guidelines stipulated by the Preferred Reporting Items for Systematic Reviews and Meta-Analysis (PRISMA) (Page et al., 2021), ensuring a methodologically sound and transparent approach. Moreover, the study protocol received registration with the International Prospective Register of Systematic Reviews (PROSPERO), CRD42023494163.

### Search strategy and study selection

A systematic search of the literature for pertinent published studies was performed in electronic databases, including China National Knowledge Infrastructure (CNKI), Embase, PubMed, Wan Fang Database, and Web of Science. The preliminary search was executed in September 2023, and a subsequent search was carried out in November 2023. Retrieved potential keywords by reviewing literature reviews, consulting experts, and combined relevant keywords in different ways utilizing operators “AND” “OR” and “NOT” to combine relevant keywords in various ways: concurrent resistance, combined strength, combined aerobic, concurrent endurance, concurrent training, combined training, irisin, and FNDC5. A detailed search syntax is provided in the Table S1. Furthermore, to ensure a thorough retrieval of relevant literature, a meticulous monitoring process was implemented. This involved tracking citations within the included articles, scrutinizing pertinent literature reviews, and surveying meta-analyses to identify potential studies that contribute to the comprehensiveness of the gathered evidence base. The two authors (Y.C. and J.M.) independently screened articles and resolved disagreements through consultation with a third author, S.B.

### Eligibility criteria

Studies relevant to this meta-analysis were included based on pre-established PICOs principles (Richardson et al., 1995). Studies conducted in either English or Chinese were deemed eligible for inclusion if they met the following criteria:

(1) Population—adult participants aged 18 years or above with a body mass index (BMI) equal to or exceeding 25 (for Asian populations, greater than or equal to 23) were included in this study, encompassing both general and clinical populations.

(2) Intervention—performing both aerobic and resistance training on the same day or alternating days (not conducted as acute training).

(3) Comparator—participants were instructed not to alter their existing lifestyle.

(4) Outcomes—circulating irisin levels were measured using Enzyme-Linked Immunosorbent Assay (ELISA) at least 12 h post-exercise.

(5) Study type—were RCTs.

After eliminating duplicates, two authors (Y.C. and J.M.) assessed titles, abstracts, and full texts to identify articles for inclusion. Inconsistencies were addressed through consultation with the third author (S.B.).

### Risk of bias and quality assessment

The risk of bias was assessed according to the Cochrane Handbook (Rob1), which consisted of six domains: random sequence generation, allocation concealment, double blinding of participants and personnel, blinding of outcome assessment, incomplete reporting of outcome, and other bias (Sterne et al., 2019). To ensure methodological rigor, two evaluators (Y.C. and J.M.) independently assessed each potential bias. In instances where disparities in assessments arose between the two evaluators, resolution was achieved through a deliberative consensus process, involving a third author (S.B).

For enhanced transparency and comprehension of the risk of bias across the reviewed literature, this study utilized Stata 16 and Review Manager 5.4 software to generate visual representations of the risk of bias assessments.

### Data extraction

Two reviewers (Y.C. and J.M.) autonomously retrieved the subsequent data: author, year of publication, participant characteristics (age, gender, sample size, and health status), as well as comprehensive details on training variables, including intervention duration, frequency, intensity, and the total intervention period. Additionally, the primary outcome measures were systematically collected. In instances where discrepancies in data extraction emerged, a resolution mechanism was implemented through consultation with a third author (S.B.), ensuring consensus and clarity.

The mean and standard deviation (SD) of post-exercise irisin were extracted to calculate the effect size. In instances where data were found to be absent, diligent efforts were made to procure the necessary information by reaching out to the respective researchers involved in the study. Articles or study results failing to provide the requested data, whether due to non-response, refusal, or inherent omissions, were subject to exclusion. In cases where data were solely presented graphically, extraction was executed utilizing the GetData Graph Digitizer software (Gao & Yu, 2023).

### Data synthesis and statistical analysis

Due to the utilization of various ELISA brands for irisin level measurements and considering the differences in irisin measurement among different brands (Pekkala et al., 2013), this analysis computed the standardized mean difference (SMD), expressed as Cohen’s d, along with 95% confidence intervals, utilizing either a random or fixed-effects model. The choice between models was contingent upon the degree of heterogeneity. Heterogeneity was meticulously assessed through forest plots and the *I*^2^ test, where values of 25%, 50%, and 75% indicated low, moderate, and high heterogeneity, respectively. When *I*^2^ equals or exceeds 50%, it suggests the presence of substantial heterogeneity; therefore, a random-effects model is used. Conversely, a fixed-effects model is used when *I*^2^ is below 50% (Higgins et al., 2003). According to the recommendations of Cohen (1988), an effect size of approximately 0.4 suggests a small effect, around 0.4–0.7 indicates a moderate effect, and approximately 0.7 signifies a significant effect.

Cochrane *Q* Test was used to assess heterogeneity within each group, with a significance level set at a *p*-value less than 0.1, while the I-squared (*I*^2^) statistic quantified the proportion of variation across studies attributed to heterogeneity rather than random error. Additionally, Cochran’s *Q* Test was applied to examine differences between subgroups, and the *I*^2^ statistic was used to measure the extent of heterogeneity between these subgroups (Borenstein, 2024). Sensitivity analyses were conducted to evaluate the impact of each study on the overall effect size by iteratively removing each study from the model. Funnel plots and Egger’s regressions were instrumental in identifying potential sources of publication bias. Due to the limited number of included studies, predetermined categorical variables (such as gender, health status, and total intervention period) were used to identify sources of heterogeneity, and meta-regression analysis was not conducted. All statistical analyses were carried out using Stata 16.0 and Review Manager 5.4 software. Significance levels were set at a double-sided *p* < 0.05 unless otherwise specified.

Two authors (Y.C. and J.M.) independently assessed the certainty of the results using the Grading of Recommendations Assessment, Development, and Evaluation (GRADE) system. The levels of certainty are categorized as high, moderate, low, and very low. Initially, since all included studies are RCTs, they are rated as “high” quality. However, the certainty of the results may be downgraded based on the following considerations: risk of bias, inconsistency, indirectness, imprecision, and other considerations (Guyatt et al., 2008).

## Results

**Figure 1:**
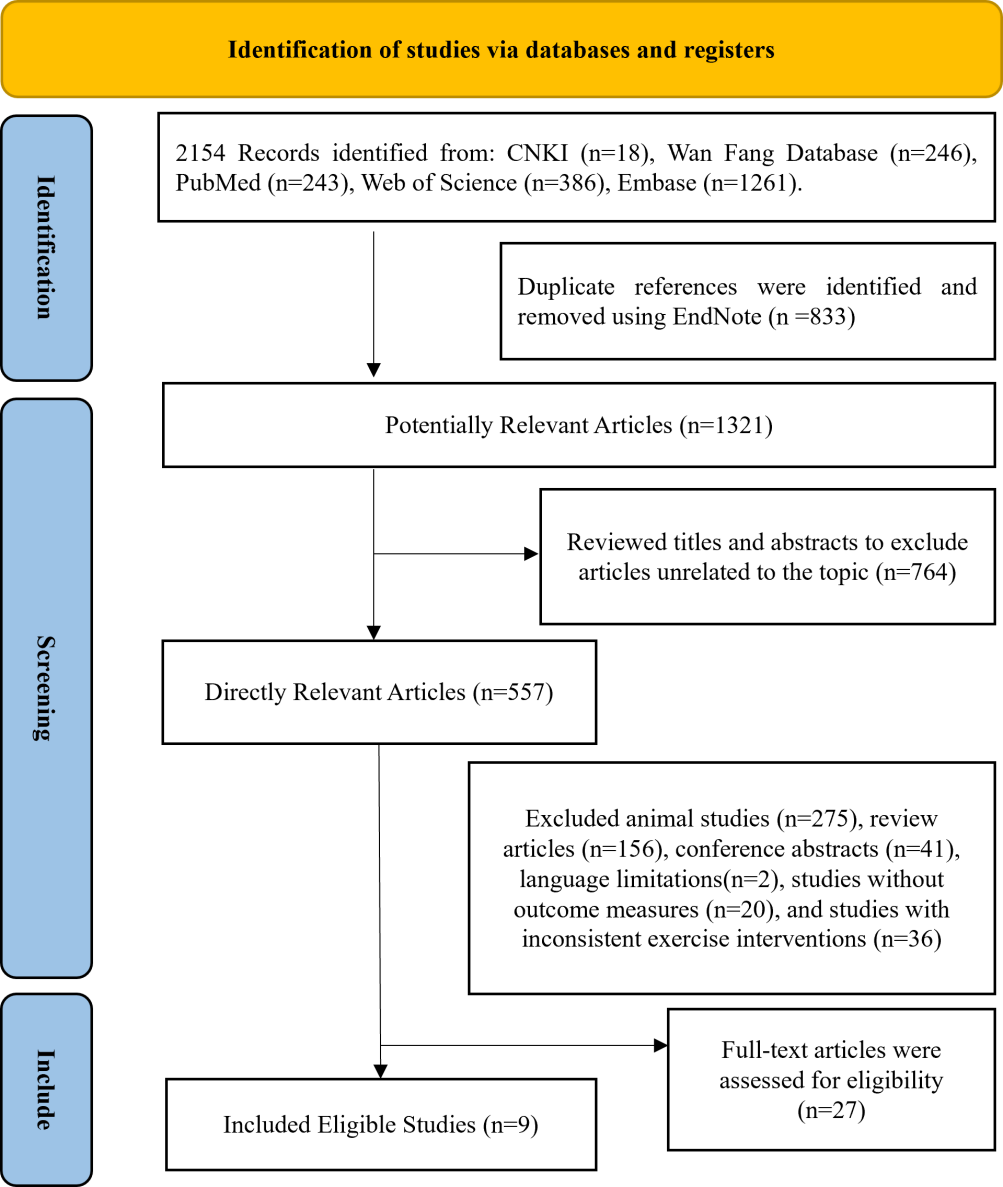
Following the PRISMA statement’s literature selection flowchart.

The process of screening literature for this systematic review is illustrated in Fig. 1. By searching five databases, a total of 2,154 studies were initially recognized as potentially suitable for analysis, 1,321 studies remained after eliminating duplicates, 557 articles remained after screening titles and abstracts, followed by the exclusion of animal experiments, reviews, conferences, language limitations, studies lacking clear outcome measures, and those with inconsistent exercise protocols, 27 studies were included in the full-text review, and ultimately nine eligible articles (Amanat et al., 2020; Banitalebi et al., 2019; Dianatinasab et al., 2020; Ghodrati et al., 2023; Makiel et al., 2023; Merawati et al., 2023; Chen, 2023; Rad et al., 2023; Rashti et al., 2019) were included in this meta-analysis.

### Characteristics of the included studies

The analysis comprised nine randomized controlled trials, encompassing a total of 264 participants. Of these, 153 underwent the exercise intervention, while 111 served as controls, with ages ranging from 20 to 70 years. All studies incorporated both intervention and control groups with individuals classified as overweight or obese. The selected studies encompassed participants with overweight or obesity (*n* = 9), MetS (*n* = 3) (Amanat et al., 2020; Dianatinasab et al., 2020; Makiel et al., 2023), T2DM (*n* = 3) (Banitalebi et al., 2019; Ghodrati et al., 2023; Rad et al., 2023).

The AT was performed by treadmill, bicycle ergometer, or group gymnastics. All studies employed maximum heart rate (HRmax) as a metric for monitoring AT intensity. In each of the nine RCTs, RT were integrated, including exercises such as deep squats, bench presses, and leg presses. Reported intensities for AT ranged from 50–95% of HRmax, and for RT, they ranged from 40–85% of one-repetition maximum (1RM). The CT training duration ranged from 50 to 80 min, conducted 2 to 4 times per week, with a total intervention period spanning 4 to 24 weeks.

All included studies provided data for the effect of CT on circulating irisin levels, utilizing commercially available ELISA Kit from different regions. Eight articles analyzed irisin concentration in serum, while another did not specify the medium (Merawati et al., 2023). Measurement times for irisin levels ranged from 12 to 48 h after exercise across the included studies. Table 1 provides a comprehensive summary of the characteristics of the included studies.

### Quality of evidence assessment

**Figure 2:**
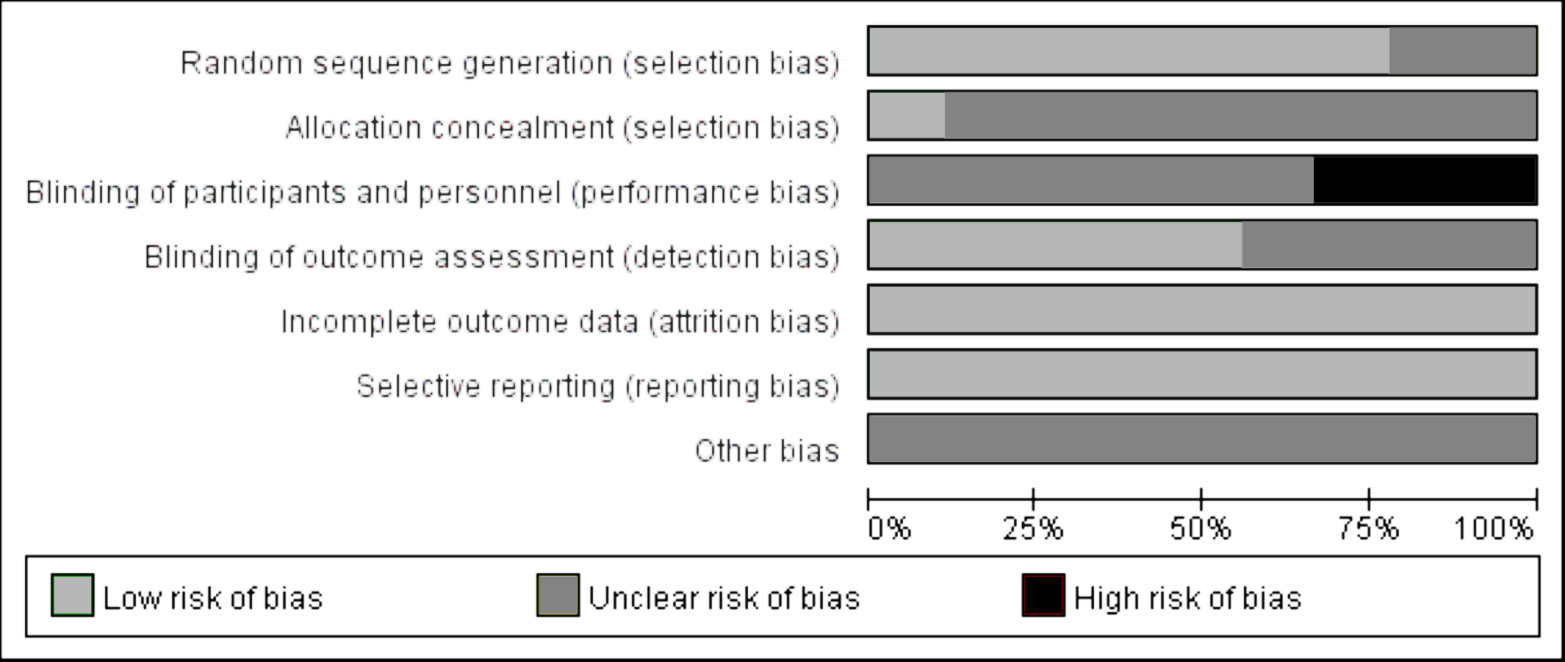
Evaluation of literature quality on the effects of concurrent training on circulating irisin levels in overweight or obese individuals.

The publication quality and bias risk assessment in the included studies were conducted utilizing the Cochrane Risk of Bias tool, and the findings are visually depicted in Fig. 2. The distribution of the percentage risk of bias across different domains is as follows: for the generation of randomized sequences, 77% were classified as low risk, 33% as unclear risk, and 0% as high risk. Allocation concealment exhibited a distribution of 11% low risk, 89% unclear risk, and 0% high risk. Double blinding of participants and personnel revealed 0% low risk, 67% unclear risk, and 33% high risk. In terms of blinding of outcome assessment, the distribution was 56% low risk, 44% unclear risk, and 0% high risk. Both incomplete reporting of outcome and selective reporting demonstrated 100% low risk. Finally, the category of other bias was characterized by 100% uncertainty (unclear risk).

**Table 1:** Characteristics of the included studies. (Dianatinasab et al., 2020; Rashti et al., 2019; Merawati et al., 2023; Banitalebi et al., 2019; Makiel et al., 2023; Rad et al., 2023; Ghodrati et al., 2023; Amanat et al., 2020; Chen, 2023).

Study	Measurement	Serum/ Plasma	Measurement brand	Training background	Gender	Age	Concurrent training	Same session/ Alternating session	Sequence	Control	Frequency	Training time per training session	Number of weeks
Dianatinasab et al. (2020)	ELISA (24 h after exercise)	Serum	Phoenix Pharmaceuticals^®^ (Cat. No.: Not specified)	Overweight or obesity comorbid with metabolic syndrome	female	53.47 ± 6.53	1. running on the treadmill, Duration:30 min, HRmax:60%–75% 2. resistance training:1 sets of 10 different exercises,1RM:60–80% Duration:30 min (*n* = 13)	Same	AR	(*n* = 15) Not to change their physical activity	2–3	60 min	8
Rashti et al. (2019)	ELISA (12 h after exercise)	Serum	BioVendor^®^ (Cat. No.: Not specified)	Overweight or obesity	female	54.8 ± 4.17	1. HIIT: 4 × 4 times at 85–95% HRmax,4-minute recovery period at 65% HR max, 40–50 min 2. resistance training:3-4 sets of 9 different exercises with 8-15 repetitions, 1RM: 40-85%, 35-45 min (*n* = 15)	Alternating	N	(*n* = 10) Not to change their physical activity	3	50–65 min	10
Merawati et al. (2023)	ELISA (24 h after exercise)	N	Elabscience^®^ (Cat. No.: E-EL-H6120)	Overweight or obesity	female	20–22	1. resistance training sets of 10 different exercises with 12 repetitions 1RM:60–70% 2. running at 60–70%HRmax in the same session the duration of the resistance training sessions was 40 min, followed by 40 min for aerobic training (*n* = 9)	Same	RA	(*n* = 7) Not to change their physical activity	3	80 min	4
Banitalebi et al. (2019)	ELISA (48 h after exercise)	Serum	Hangzhou Eastbiopharm Co^®^ (Cat. No.: KE90905)	Overweight or obesity combined with type 2 diabetes	female	55 ± 6	1. running on the treadmill or ergometer, Duration: 20-30 min, HRmax:50%–70% 2. resistance training:2–3 sets of 10-12 repetitions, 1RM:60–80% (*n* = 14)	Same	AR	(*n* = 14) not to change their physical activity	3	50 min	10
Makiel et al. (2023)	ELISA (24 h after exercise)	Serum	Shanghai Sunred Biological Technology Co.^®^ (Cat. No.: 201-12-5328)	Overweight or obesity comorbid with metabolic syndrome	male	36.6 ± 6.9	1. resistance training:3 sets of 6-9 different exercises, 1RM:50–70%, duration of 30–40 minutes 2. followed by 10–20 min for aerobic training, running/walking on the treadmill at 50%HRmax in the same session (*n* = 16)	Same	RA	(*n* = 15) Not to change their physical activity	3	60 min	12
Rad et al. (2023)	ELISA (12 h after exercise)	Serum	ZellBio GmbH^®^ (Cat. No.: Not specified)	Overweight or obesity combined with type 2 diabetes	male	40–50	1. HIIT: running on the treadmill, duration:1 min, HRmax:75%–95% 2. resistance training:3 sets of 6 different exercises with 8-18 repetitions, 1RM:40–80% (*n* = 15)	Same	RA/AR	(*n* = 13) Not to change their physical activity	3	N	12
Ghodrati et al. (2023)	ELISA (12 h after exercise)	Serum	Hangzhou EastBiopharm Co.^®^ (Cat. No.: Not specified)	Overweight or obesity combined with type 2 diabetes	female	57.52 ± 5.07	1. running on the bicycle ergometer, duration: 20 min, HRmax:55%–75% 2. resistance training:3 sets of 6–12 max 1RM:65–85% (*n* = 12)	Same	AR	(*n* = 9) Not to change their physical activity	3	65 min	12
Amanat et al. (2020)	ELISA (12 h after exercise)	Serum	Phoenix Pharmaceuticals^®^ (Cat. No.: Not specified)	Overweight or obesity comorbid with metabolic syndrome	female	54.5 ± 6.9	1. running on the treadmill, duration:30 min, HRmax:60%–75% 2. resistance training:1 sets of 10 different exercises, 1RM:60–80%, duration:30 min (*n* = 13)	Same	AR	(*n* = 14) Not to change their physical activity	3	60 min	12
Chen (2023)	ELISA(N)	Serum	Shanghai Hengyuan Technology Co., Ltd.^®^ (Cat. No.: Not specified)	Overweight or obesity	female	50–70	1. Aerobic exercise entails group gymnastics sessions, maintaining heart rates within the range of 55–65% of HRmax. 2. Resistance training involves elastic band exercises initially, transitioning to adjustable weight equipment in the gym after 12 weeks, with a single exercise session lasting approximately 60 min and intensity set at 50–85% of 1RM (*n* = 15)	Alternating	N	(*n* = 14) Not to change their physical activity	4	60 min	24

**Notes.**

ELISA Enzyme-linked immunosorbent assay HRmax Maximum heart rate RM Repetition maximum HIIT High-Intensity Interval Training AR Concurrent training with aerobic exercise preceding resistance exercise RA Concurrent training with resistance exercise preceding aerobic exercise Same Concurrent training is conducted in the same session Alternating Concurrent training is conducted in the alternating session N Not report

### Meta-analyses

**Figure 3:**
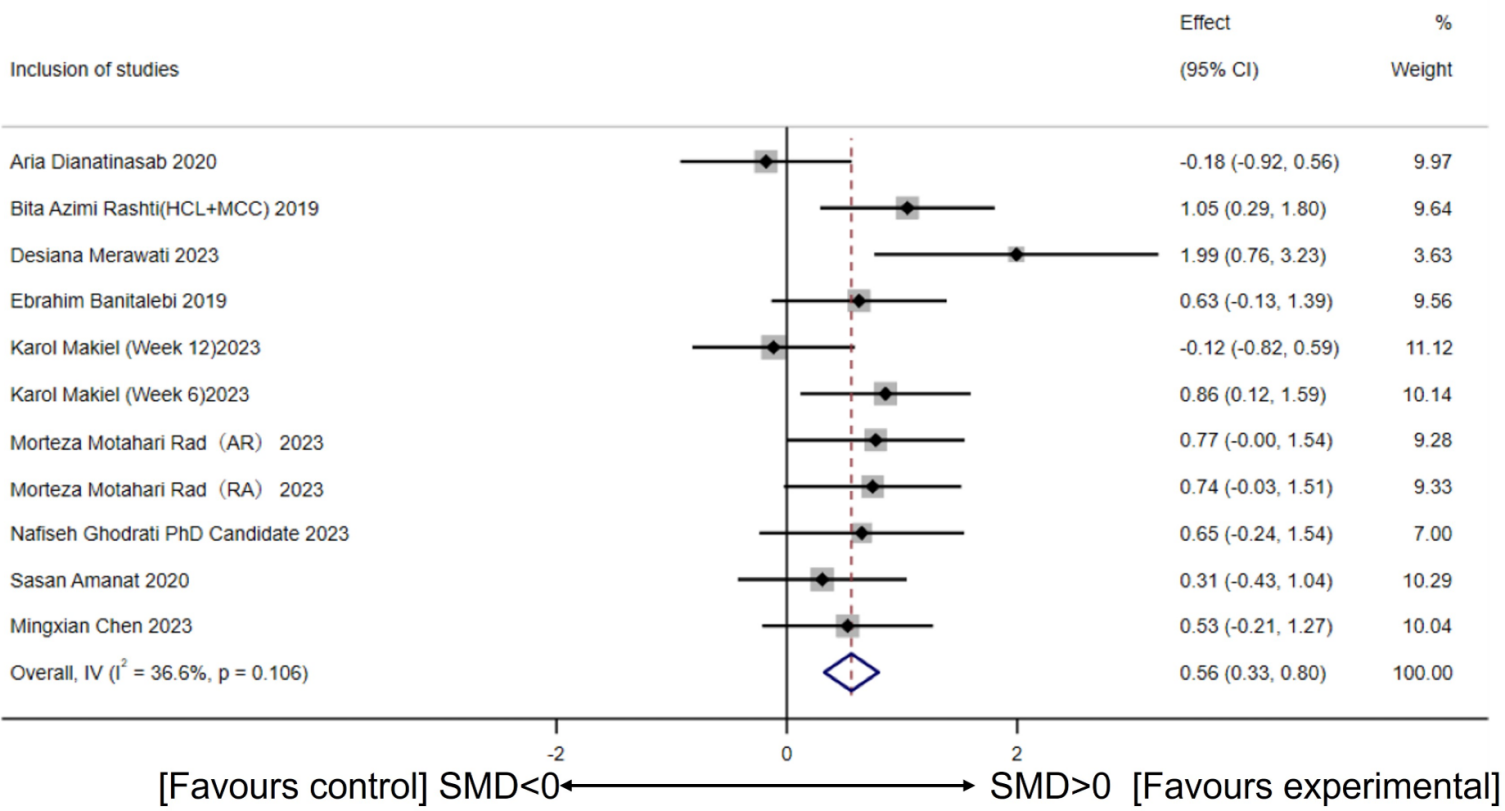
Meta-analysis forest plot of concurrent training improving circulating irisin levels in overweight or obese individuals. HCI, High-intensity concurrent interval exercise; MCC, Moderate-intensity continuous concurrent exercise; AR, Concurrent training with aerobic exercise preceding resistance exercise; RA, Concurrent training with resistance exercise preceding aerobic exercise; SMD, Standardized mean difference; 95CI, 95% Confidence Interval. (Dianatinasab et al., 2020; Rashti et al., 2019; Merawati et al., 2023; Banitalebi et al., 2019; Makiel et al., 2023; Rad et al., 2023; Ghodrati et al., 2023; Amanat et al., 2020; Chen, 2023).

Based on 11 intervention groups involving 264 participants (from nine studies), the heterogeneity test showed acceptable variability (*I*^2^ = 36.6%, *p* for heterogeneity = 0.106). A fixed-effect model revealed that CT intervention significantly increased circulating irisin levels compared to the control group (SMD = 0.56, 95% CI [0.33–0.80], *p* = 0.00) (Fig. 3).

**Figure 4:**
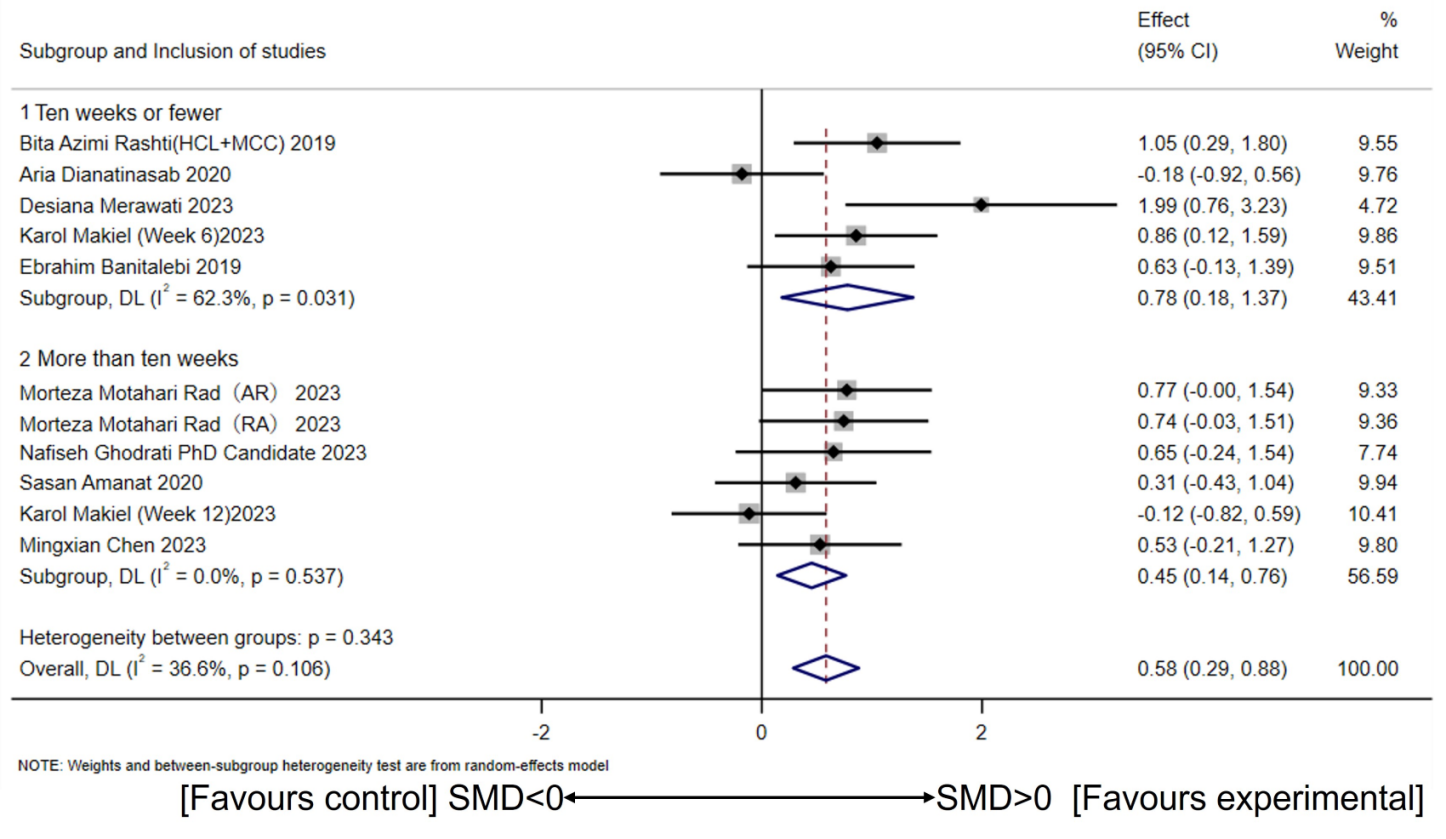
Subgroup analysis of concurrent training on circulating irisin levels in individuals with different intervention periods. (Dianatinasab et al., 2020; Rashti et al., 2019; Merawati et al., 2023; Banitalebi et al., 2019; Makiel et al., 2023; Rad et al., 2023; Ghodrati et al., 2023; Amanat et al., 2020; Chen, 2023).

Based on studies indicating that CT exceeding 10 weeks interferes with muscle mass and affects irisin secretion, subgroup analysis was conducted using a 10-week cutoff (Fyfe, Bishop & Stepto, 2014; Hickson, 1980). The random-effects model revealed a large effect size for CT interventions of 10 weeks or less (SMD = 0.78, 95% CI [0.18–1.37], *p* = 0.01; *I*^2^ = 62.3%, *p* for heterogeneity = 0.03) and a moderate effect size for CT interventions exceeding 10 weeks (SMD = 0.45, 95% CI [0.14–0.76], *p* = 0.00; *I*^2^ = 0%, p for heterogeneity = 0.53) (Fig. 4). No significant between-group differences were observed (*I*^2^ =0%, *p* = 0.34).

**Figure 5:**
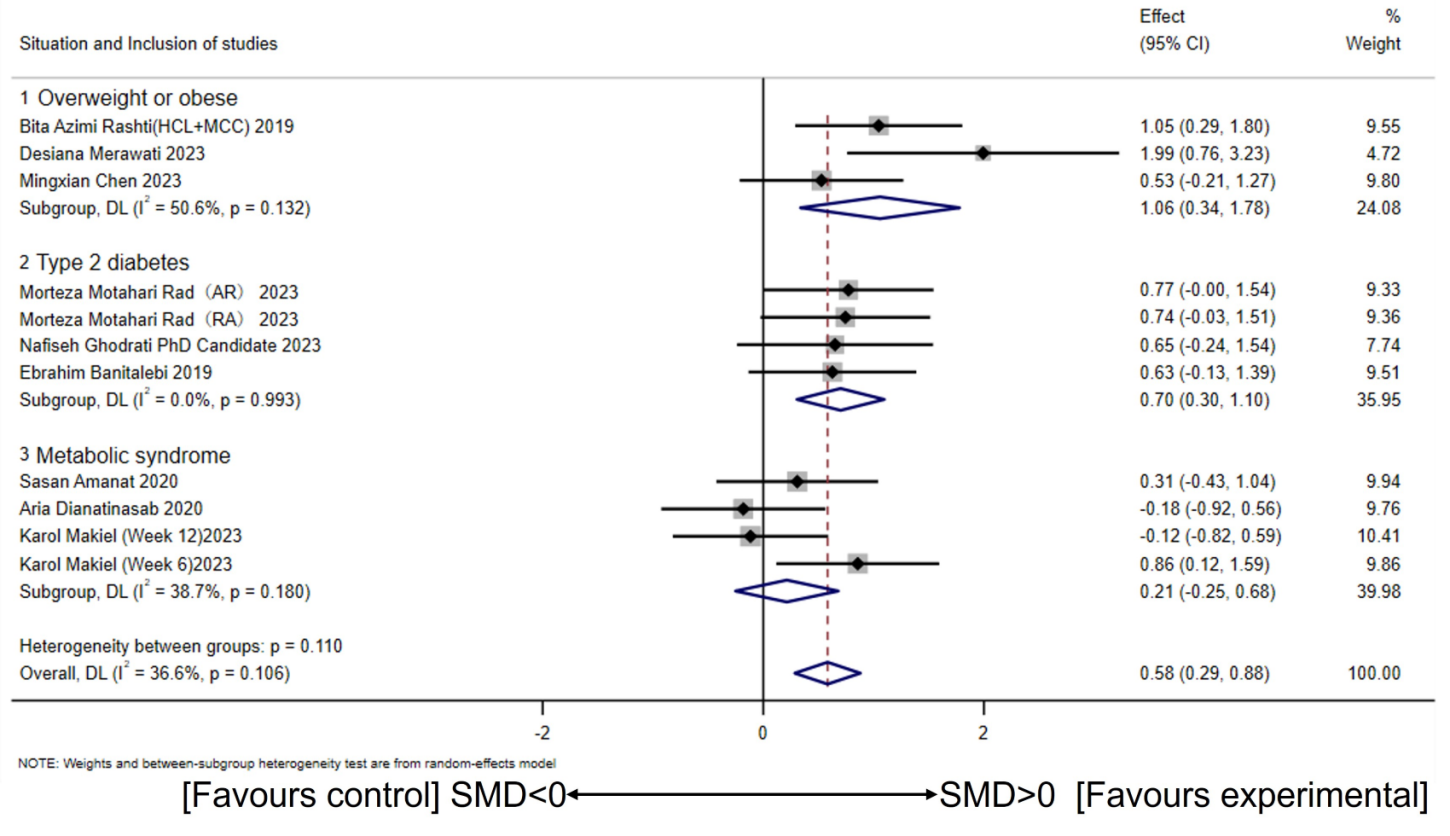
Subgroup analysis of concurrent training on circulating irisin levels in individuals with different health conditions. (Dianatinasab et al., 2020; Rashti et al., 2019; Merawati et al., 2023; Banitalebi et al., 2019; Makiel et al., 2023; Rad et al., 2023; Ghodrati et al., 2023; Amanat et al., 2020; Chen, 2023).

Using a random-effects model, subgroup analysis based on health conditions revealed significant increases in circulating irisin levels among overweight or obese participants (a large effect size) (SMD = 1.06, 95% CI [0.34–1.78], *p* = 0.00; *I*^2^ = 50.6%, p for heterogeneity = 0.13). Similarly, overweight or obese participants with T2DM also showed significant increases in circulating irisin levels (a large effect size) (SMD = 0.70, 95% CI [0.30–1.10], *p* = 0.00; *I*^2^ = 0.0%, *p* for heterogeneity = 0.99). However, CT did not affect circulating irisin levels in overweight or obese participants with MetS (SMD = 0.21, 95% CI [−0.25–0.68], *p* = 0.36; *I*^2^ = 38.7%, *p* for heterogeneity = 0.18) (Fig. 5). In addition, there were significant between-group differences (*I*^2^ = 53.9%, *p* = 0.11).

Based on age-stratified subgroup analysis, CT significantly increased circulating irisin levels in the 45–60 age group, while no significant effect was observed in other age groups. In addition, subgroup analysis based on post-exercise measurement time demonstrated that circulating irisin levels were significantly increased when measured 12 h after exercise, whereas there was no significant effect observed at other measurement times. Furthermore, subgroup analyses based on gender, CT sequence, and session did not reveal highly significant effects. Finally, none of the above categorical variables showed significant between-group differences (Table 2).

Visual inspection of the funnel plot revealed asymmetry in the lower right corner, suggesting a potential publication bias (Fig. S1). Additionally, the Egger’s test results supported this finding (*p* = 0.03) (Fig. S2). Subsequent application of the trim-and-fill method demonstrated consistent results before and after, indicating that potential missing studies did not exert a significant influence on the overall outcome (Fig. S3). Sensitivity analyses demonstrated that the results were stable and not significantly influenced by any individual study (Fig. S4).

The results of the GRADE assessment indicate that due to the risk of bias and the small sample size, the study’s rating has been downgraded to low (Table S2).

## Discussion

Despite the high heterogeneity and overall lower quality of the included literature in previous studies, they have contributed to understanding the effects of exercise interventions on irisin secretion and health promotion (Jandova et al., 2021; Torabi et al., 2024). To our knowledge, this study represents the pioneering effort to scrutinize the influence of short- and long-term CT on the levels of circulating irisin among overweight or obese individuals through a comprehensive meta-analytical approach. The findings of this meta-analysis reveal a discernible augmentation in circulating irisin levels induced by short- and long-term CT when contrasted with control groups, displaying a moderate effect. It is noteworthy that despite the interference effects of CT, which suggest a decline in muscle strength and mass with AT interventions lasting more than 10 weeks, our study indicates that this scenario did not affect irisin levels. Subgroup analyses, stratified by health status, underscore the efficacy of short- and long-term CT in enhancing circulating irisin levels, particularly in the demographic of overweight or obese individuals and those afflicted by T2DM, offering a potential avenue for ameliorating these health conditions. Conversely, for individuals with MetS, it appears necessary to explore alternative strategies. Furthermore, irisin levels may also be influenced by age and the timing of measurements post-exercise.

**Table 2:** Subgroup analysis of age, gender, sequence, session, and post-exercise irisin measurement time.

Categorical variable	Study quantity	Sample size	Model	Subgroup	The within-group effect				The between-group differences	
					Heterogeneity		Effect(95%Cl)	*p*		
					*I* ^2^	*p*			*I* ^2^	*p*
	3	47	Random	<45	78.6%	0.01	0.81(−0.26,1.89)	0.13		
Age	7	188		45–60	6.50%	0.38	0.56(0.25,0.86)	0.00	0.00%	0.91
	1	29		>60	one arm					
Gender	4	74	Random	Male	35.50%	0.20	0.55(0.08,1.01)	0.02	0.00%	0.86
	7	190		Female	45.90%	0.09	0.62(0.20,1.03)	0.00		
	4	75		AR	0.00%	0.42	0.41(0.07,0.76)	0.01		
Sequence	5	121	Random	RA	68.20%	0.02	0.76(0.03,1.50)	0.04	0.00%	0.46
	2	68		N	one arm					
Session	9	196	Random	Same session	42.9%	0.08	0.54(0.19,1.89)	0.00	0.00%	0.44
	2	68		Alternating session	0.00%	0.34	0.78(0.25,1.31)	0.00		
Measurement time	5	132	Random	ELISA (12 h after exercise)	0.00%	0.74	0.70(0.35,1.05)	0.00	0.00%	0.96
	4	75		ELISA (24 h after exercise)	75.50%	0.01	0.54(−0.29,1.37)	0.21		
	1	28		ELISA (48 h after exercise)	one arm							
	1	29		N						

**Notes.**

ELISA Enzyme-linked immunosorbent assay Random Random effects model AR Concurrent training with aerobic exercise preceding resistance exercise RA Concurrent training with resistance exercise preceding aerobic exercise Same Concurrent training is conducted in the same session Alternating Concurrent training is conducted in the alternating session

The results of this meta-analysis suggest that short- and long-term CT moderately increases circulating irisin levels in overweight or obese individuals compared to the control group. This result aligns with findings from a prior RCT (Amanat et al., 2020). CT involves aerobic and anaerobic energy systems, combining strength/power and AT (Markov et al., 2023). This type of training may impact irisin concentration in following two primary ways. Firstly, CT may enhance the mammalian Target of Rapamycin (mTOR) signaling pathway in type II muscle fibers, leading to increased muscle mass and hypertrophy (Fyfe, Bishop & Stepto, 2014). Furthermore, previous evidence showed that irisin, as a myokine, was positively correlated with muscle mass (Aladag, Mogulkoc & Baltaci, 2023; Kim et al., 2016). Secondly, adding ET to RT amplifies the signaling expression of PGC-1α in type I muscle fibers (Baar, 2006), creating favorable conditions for downstream irisin formation (Zuo et al., 2023). Immunohistochemical analysis reveals that irisin is located extracellularly between muscle fibers (Perakakis et al., 2017). Consequently, additional RT during ET has been suggested to increase creatine kinase (CK), inducing muscle damage and further elevated circulating irisin levels (Huh et al., 2015). However, a RCT study demonstrated inconsistent results (Dianatinasab et al., 2020). These inconsistencies may arise due to the measurement timing of irisin post-exercise, as irisin is a fast-degrading myokine (Parada-Sánchez et al., 2022). It is noteworthy that the population in the aforementioned RCT consists of individuals with Mets. Therefore, variability in study populations may also lead to inconsistencies. Furthermore, irisin was measured using various commercially available ELISA kits, and their accuracy has recently been disputed (Maak et al., 2021; Perakakis et al., 2017; Qiu et al., 2018).

Moreover, despite concurrent aerobic and anaerobic physiological adaptations achievable through CT, interference effects are present. As noted by Hickson, after the tenth week of CT, muscle strength declines while aerobic capacity continues to steadily improve (Hickson, 1980). The interference effect primarily manifests in the compromised resistance adaptation of CT compared to RT alone (Fyfe, Bishop & Stepto, 2014; Murlasits, Kneffel & Thalib, 2018). Some researchers concluded that the addition of ET to RT may lead to a reduction in muscle mass (Lundberg et al., 2022) and impair muscle strength, consequently diminishing irisin secretion (Aladag, Mogulkoc & Baltaci, 2023; Ma et al., 2021). Mechanistically, the (mechanistic) target of the mammalian target of rapamycin complex 1 (mTORC1) has been identified as a critical mediator in the augmentation of protein synthesis induced by RT and the prevention of muscle atrophy (Bodine et al., 2001). In contrast to the RT signaling pathways described above, ET also induces a variety of signaling cascades. For instance, it has been demonstrated that activation of AMP-activated protein kinase (AMPK) inhibits mTORC1 and its downstream signaling pathways (Bolster et al., 2002). Additionally, a negative correlation between AMPK phosphorylation and muscle hypertrophy has been observed, suppressing protein synthesis, and favoring aerobic adaptation (Thomson, Fick & Gordon, 2008). Contrary to the aforementioned study, our research did not find evidence of interference effects from CT on circulating irisin levels. Consistently, another study indicated that a 12-week CT intervention increased circulating irisin and follistatin (FST) while decreasing MSTN (Rad et al., 2023) in patients with T2DM (Rad et al., 2023). Considering that the majority of prior investigations have been carried out in animal models, it requires further clarification whether CT “interferes” with the regulation of human irisin levels as the duration of training extends (Erickson, 2013).

Our study reveals that CT improves circulating irisin levels in overweight or obese individuals, including those with T2DM. Previous research has suggested that obese individuals without T2DM experience enhanced FNDC5 synthesis and subsequent irisin release, potentially contributing to the regulation of glucose retention in muscles and preventing hyperglycemia and obesity (Aladag, Mogulkoc & Baltaci, 2023; Perakakis et al., 2017). While irisin levels are significantly elevated in the obese population compared to normal weight individuals (Löffler et al., 2015), they are found to decrease during bariatric surgery, indicating a correlation between body fat and irisin levels (Yu et al., 2019). However, despite the potential decrease in irisin due to prolonged CT to reduce body fat, an increase in muscle mass may counteract this negative effect (Makiel et al., 2023), given that muscle tissue is a primary producer of irisin (Roca-Rivada et al., 2013). Interestingly, a study reported that circulating irisin was not associated with BMI, body fat, and muscle mass (Yan et al., 2014). T2DM is a systemic metabolic disorder marked by insulin resistance and inadequate insulin secretion, constituting 90% of all diabetes types (Yang et al., 2019). A CT study in 21 older participants with T2DM found that irisin did not change significantly after 12 weeks of the training intervention (Ghodrati et al., 2023). However, it was worth noting that the control group in this study showed a significant decrease in irisin levels, highlighting the potential of CT to maintain circulating irisin levels in individuals with T2DM (Bonfante et al., 2017a; Ghodrati et al., 2023). Furthermore, patients with T2DM are found to have lower circulating irisin levels (Liu et al., 2013; Yan et al., 2014), possibly due to the increased demand for sustaining glucose homeostasis and ameliorating insulin resistance in these individuals (Aladag, Mogulkoc & Baltaci, 2023). Therefore, compared to overweight or obese individuals alone, the effect of exercise training on irisin levels may have been attenuated in individuals with T2DM.

The MetS is intricately linked with conditions of obesity and T2DM, exhibiting a constellation of interrelated metabolic disorders such as insulin resistance, atherosclerotic dyslipidemia, visceral obesity, and hypertension (Makiel et al., 2023). A previous self-controlled trial demonstrated that HIIT, Moderate-Intensity Continuous Training (MICT), or RT significantly increased circulating irisin levels 1 h after exercise, regardless of whether the individual had MetS or not (Huh et al., 2015). This implies that exercise training can increase irisin of participants with MetS independently of the type of exercise. Another study showed that acute strenuous exercise can modulate circulating irisin levels, highlighting exercise intensity as a regulatory factor for irisin levels (Daskalopoulou et al., 2014; Löffler et al., 2015). On the contrary, this meta-analysis reveals that short- and long-term CT does not improve circulating irisin levels in individuals with MetS. This suggests that the responses induced by acute exercise might not translate into long-term adaptations in circulating irisin for individuals with MetS. It was worth noting that participants with MetS have higher levels of circulating irisin as well as irisin resistance (Werida, El-Gharbawy & Mostafa, 2021). Consequently, the intervention aimed at reducing irisin concentrations (ameliorating irisin resistance) may explain the lack of change in circulating irisin concentrations (Makiel et al., 2023).

Finally, additional factors that need to be considered include the age of the subjects and the timing of irisin measurement post-exercise. Torabi et al. (2024) indicates that exercise interventions do not affect circulating irisin in individuals over 40 years old. Contrary to our findings, our study demonstrates that short- and long-term CT significantly improves circulating irisin levels in individuals aged 45–60. However, no significant differences were observed between the different age subgroups. This discrepancy may primarily be attributed to differences in the health status of the subjects included in the studies and the various exercise protocols employed. It is noteworthy that in our study, the timing of irisin measurement post-exercise indicated a significant increase in circulating irisin levels at 12 h post-exercise, with no significant effects observed at other time points. Similarly, the analysis revealed that there were no significant differences between the groups. This observation suggests a potential exercise arousal effect. In contrast to our findings, another study reported elevated irisin levels 72 h after 10 repeated 40-meter sprints, possibly linked to higher oxidative stress, muscle damage, and satellite cell activation (Cosio et al., 2023). This inconsistency may be attributed to differences in the exercise protocols used and the health status of the participants in that study.

Nevertheless, it is imperative to recognize and address the limitations of this meta-analysis. Firstly, the nature of exercise training poses a challenge to the implementation of a double-blind experimental design, given its practical constraints. Secondly, while all included studies were randomized controlled trials, the relatively small sample sizes within the studies may limit the generalizability of our findings. Further, the studies included in this research have inconsistent timing for measuring irisin post-intervention. This inconsistency might confound the effects of acute and chronic exercise on circulating irisin levels (arousal effects of exercise), necessitating further clarification in future studies. Additionally, due to the absence of a standardized criterion for CT intensity across the included studies, conducting subgroup analysis becomes challenging. Consequently, further research is warranted to explore and elucidate the effects of varying intervening variables of CT on circulating irisin levels, offering a more comprehensive understanding of this complex relationship.

## Conclusions

Short- and long-term concurrent training can effectively improve circulating irisin levels in overweight or obese individuals. However, the effects of short- and long-term concurrent training should consider the participants’ health status, age, and the timing of post-exercise measurements to maximize health benefits. This discovery is especially significant for overweight or obese individuals with chronic diseases. To gain a better comprehensive understanding, additional meticulously designed randomized controlled trials are imperative to investigate the impacts of various concurrent training variables on circulating irisin levels in different populations, as well as the underlying mechanisms.

## Supplemental Information

10.7717/peerj.17958/supp-1 Supplemental Information 1 The search strategies utilized to retrieve relevant studies from various databases for this systematic review and meta-analysis This outlines the specific search terms and Boolean operators used to ensure comprehensive coverage of the topic.

10.7717/peerj.17958/supp-2 Supplemental Information 2 GRADE evidence profile CI, confidence interval;
SMD, Standardized Mean Difference;
CT, concurrent exercise;
Con, control;
^a^: Implementing a double-blind randomized controlled trial for exercise interventions is challenging, which may lead to a downgrading of the risk of bias assessment.
^b^: Sample size below optimal information size contributing to imprecision which lowers our certainty in effect.

10.7717/peerj.17958/supp-3 Supplemental Information 3 The funnel plot for assessing publication bias among the included studies SMD, Standardized mean difference

10.7717/peerj.17958/supp-4 Supplemental Information 4 The graphical representation of Egger’s test used to assess publication bias

10.7717/peerj.17958/supp-5 Supplemental Information 5 The outcomes of the trim-and-fill method used to address publication bias The adjusted results after accounting for potentially missing studies.

10.7717/peerj.17958/supp-6 Supplemental Information 6 The sensitivity analysis results, highlighting the robustness of the study findings The reliability of the meta-analysis results remains consistent despite the exclusion of any individual study. Lower Cl Limit, Lower Confidence Limit;
Upper Cl Limit, Upper Confidence Limit.

10.7717/peerj.17958/supp-7 Supplemental Information 7 Raw data

10.7717/peerj.17958/supp-8 Supplemental Information 8 PRISMA 2020 checklist

10.7717/peerj.17958/supp-9 Supplemental Information 9 Rationale and highlights

10.7717/peerj.17958/supp-10 Supplemental Information 10 English-language codebook

## Additional Information and Declarations

### Data Availability

The following information was supplied regarding data availability: The raw measurements are available in the Supplemental Information.
