# Supplementary material for: Short- and long-term effects of concurrent aerobic and resistance training on circulating irisin levels in overweight or obese individuals: a systematic review and meta-analysis of randomized controlled trials
Source: PeerJ. 2024 Sep 19;12:e17958. doi: 10.7717/peerj.17958 (PMC11416761; doi:10.7717/peerj.17958)
Supplement: Supplemental Information 1 — This outlines the specific search terms and Boolean operators used to ensure comprehensive coverage of the topic. [file peerj-12-17958-s001.docx]

**Supplemental Table S1**

| **PubMed (243)** | |
| --- | --- |
| #1 | ((((((((((((((((((((("Exercise"[Mesh]) OR (Exercises[Title/Abstract])) OR (Exercise, Physical[Title/Abstract])) OR (Exercises, Physical[Title/Abstract])) OR (Physical Exercise[Title/Abstract])) OR (Physical Exercises[Title/Abstract])) OR (Physical Activity[Title/Abstract])) OR (Activities, Physical[Title/Abstract])) OR (Activity, Physical[Title/Abstract])) OR (Physical Activities[Title/Abstract])) OR (Exercise, Aerobic[Title/Abstract])) OR (Aerobic Exercise[Title/Abstract])) OR (Aerobic Exercises[Title/Abstract])) OR (Exercises, Aerobic[Title/Abstract])) OR (Exercise, Isometric[Title/Abstract])) OR (Exercises, Isometric[Title/Abstract])) OR (Isometric Exercises[Title/Abstract])) OR (Isometric Exercise[Title/Abstract])) OR (Exercise Training[Title/Abstract])) OR (Exercise Trainings[Title/Abstract])) OR (Training, Exercise[Title/Abstract])) OR (Trainings, Exercise[Title/Abstract]) |
| #2 | (((((((((((((((((((((("Resistance Training"[Mesh]) OR (Strength Training[Title/Abstract])) OR (Training, Strength[Title/Abstract])) OR (Weight-Lifting Strengthening Program[Title/Abstract])) OR (Strengthening Programs, Weight-Lifting[Title/Abstract])) OR (Strengthening Program, Weight-Lifting[Title/Abstract])) OR (Weight Lifting Strengthening Program[Title/Abstract])) OR (Weight-Lifting Strengthening Programs[Title/Abstract])) OR (Weight-Lifting Exercise Program[Title/Abstract])) OR (Exercise Programs, Weight-Lifting[Title/Abstract])) OR (Exercise Program, Weight-Lifting[Title/Abstract])) OR (Weight Lifting Exercise Program[Title/Abstract])) OR (Weight-Lifting Exercise Programs[Title/Abstract])) OR (Weight-Bearing Strengthening Program[Title/Abstract])) OR (Strengthening Programs, Weight-Bearing[Title/Abstract])) OR (Strengthening Program, Weight-Bearing[Title/Abstract])) OR (Weight Bearing Strengthening Program[Title/Abstract])) OR (Weight-Bearing Strengthening Programs[Title/Abstract])) OR (Weight-Bearing Exercise Program[Title/Abstract])) OR (Exercise Programs, Weight-Bearing[Title/Abstract])) OR (Exercise Program, Weight-Bearing[Title/Abstract])) OR (Weight Bearing Exercise Program[Title/Abstract])) OR (Weight-Bearing Exercise Programs[Title/Abstract]) |
| #3 | ((((((((((((((((((concurrent exercise[Title/Abstract]) OR (concurrent training[Title/Abstract])) OR (combined training[Title/Abstract])) OR (combined exercise[Title/Abstract])) OR (combined exercise training[Title/Abstract])) OR (aerobic resistance exercise[Title/Abstract])) OR (resistance aerobic exercise[Title/Abstract])) OR (strength aerobic training[Title/Abstract])) OR (strength endurance training[Title/Abstract])) OR (resistance endurance exercise[Title/Abstract])) OR (concurrent resistance[Title/Abstract])) OR (concurrent strength[Title/Abstract])) OR (concurrent aerobic[Title/Abstract])) OR (concurrent endurance[Title/Abstract])) OR (combined resistance[Title/Abstract])) OR (concurrent training sequence[Title/Abstract])) OR (combined training sequence[Title/Abstract])) OR (concurrent training order[Title/Abstract])) OR (combined training order[Title/Abstract]) |
| #4 | #1 OR #2 OR #3 |
| #5 | ((("FNDC5 protein, human" [Supplementary Concept]) OR (fibronectin type III domain-containing protein 5 precursor, human[Title/Abstract])) OR (FRCP2 protein, human[Title/Abstract])) OR (irisin, human[Title/Abstract]) |
| #6 | #4 AND #5 |
| **Web of science (386)** | |
| #1 | (((((((((((((((((((((TS=(Exercise)) OR TS=(Exercises)) OR TS=(Exercise, Physical)) OR TS=(Exercises, Physical)) OR TS=(Physical Exercise)) OR TS=(Physical Exercises)) OR TS=(Physical Activity)) OR TS=(Activities, Physical)) OR TS=(Activity, Physical)) OR TS=(Physical Activities)) OR TS=(Exercise, Aerobic)) OR TS=(Aerobic Exercise)) OR TS=(Aerobic Exercises)) OR TS=(Exercises, Aerobic)) OR TS=(Exercise, Isometric)) OR TS=(Exercises, Isometric)) OR TS=(Isometric Exercises)) OR TS=(Isometric Exercise)) OR TS=(Exercise Training)) OR TS=(Exercise Trainings)) OR TS=(Training, Exercise)) OR TS=(Trainings, Exercise) |
| #2 | ((((((((((((((((((((((TS=(Resistance Training)) OR TS=(Strength Training)) OR TS=(Training, Strength)) OR TS=(Weight-Lifting Strengthening Program)) OR TS=(Strengthening Programs, Weight-Lifting)) OR TS=(Strengthening Program, Weight-Lifting)) OR TS=(Weight Lifting Strengthening Program)) OR TS=(Weight-Lifting Strengthening Programs)) OR TS=(Weight-Lifting Exercise Program)) OR TS=(Exercise Programs, Weight-Lifting)) OR TS=(Exercise Program, Weight-Lifting)) OR TS=(Weight Lifting Exercise Program)) OR TS=(Weight-Lifting Exercise Programs)) OR TS=(Weight-Bearing Strengthening Program)) OR TS=(Strengthening Programs, Weight-Bearing)) OR TS=(Strengthening Program, Weight-Bearing)) OR TS=(Weight Bearing Strengthening Program)) OR TS=(Weight-Bearing Strengthening Programs)) OR TS=(Weight-Bearing Exercise Program)) OR TS=(Exercise Programs, Weight-Bearing)) OR TS=(Exercise Program, Weight-Bearing)) OR TS=(Weight Bearing Exercise Program)) OR TS=(Weight Bearing Exercise Programs) |
| #3 | ((((((((((((((((((TS=(concurrent exercise)) OR TS=(concurrent training)) OR TS=(combined training)) OR TS=(combined exercise)) OR TS=(combined exercise training)) OR TS=(aerobic resistance exercise)) OR TS=(resistance aerobic exercise)) OR TS=(strength aerobic training)) OR TS=(strength endurance training)) OR TS=(resistance endurance exercise)) OR TS=(concurrent resistance)) OR TS=(concurrent strength)) OR TS=(concurrent aerobic)) OR TS=(concurrent endurance)) OR TS=(combined resistance)) OR TS=(concurrent training sequence)) OR TS=(combined training sequence)) OR TS=(concurrent training order)) OR TS=(combined training order) |
| #4 | #1 OR #2 OR #3 |
| #5 | (((TS=(FNDC5 protein, human)) OR TS=(fibronectin type III domain-containing protein 5 precursor, human)) OR TS=(FRCP2 protein, human)) OR TS=(irisin, human) |
| #6 | #4 AND #5 |
| **Embase (1261)** | |
| #1 | exercise:ab,ti OR exercises:ab,ti OR 'exercise, physical':ab,ti OR 'exercises, physical':ab,ti OR 'physical exercise':ab,ti OR 'physical exercises':ab,ti OR 'physical activity':ab,ti OR 'activities, physical':ab,ti OR 'activity, physical':ab,ti OR 'physical activities':ab,ti OR 'exercise, aerobic':ab,ti OR 'aerobic exercise':ab,ti OR 'aerobic exercises':ab,ti OR 'exercises, aerobic':ab,ti OR 'exercise, isometric':ab,ti OR 'exercises, isometric':ab,ti OR 'isometric exercises':ab,ti OR 'isometric exercise':ab,ti OR 'exercise training':ab,ti OR 'exercise trainings':ab,ti OR 'training, exercise':ab,ti OR 'trainings, exercise':ab,ti |
| #2 | 'resistance training':ab,ti OR 'strength training':ab,ti OR 'training, strength':ab,ti OR 'weight-lifting strengthening program':ab,ti OR 'strengthening programs, weight-lifting':ab,ti OR 'strengthening program, weight-lifting':ab,ti OR 'weight lifting strengthening program':ab,ti OR 'weight-lifting strengthening programs':ab,ti OR 'weight-lifting exercise program':ab,ti OR 'exercise programs, weight-lifting':ab,ti OR 'exercise program, weight-lifting':ab,ti OR 'weight lifting exercise program':ab,ti OR 'weight-lifting exercise programs':ab,ti OR 'weight-bearing strengthening program':ab,ti OR 'strengthening programs, weight-bearing':ab,ti OR 'strengthening program, weight-bearing':ab,ti OR 'weight bearing strengthening program':ab,ti OR 'weight-bearing strengthening programs':ab,ti OR 'weight-bearing exercise program':ab,ti OR 'exercise programs, weight-bearing':ab,ti OR 'exercise program, weight-bearing':ab,ti OR 'weight bearing exercise program':ab,ti OR 'weight-bearing exercise programs':ab,ti |
| #3 | 'concurrent exercise':ab,ti OR 'concurrent training':ab,ti OR 'combined training':ab,ti OR 'combined exercise':ab,ti OR 'combined exercise training':ab,ti OR 'aerobic resistance exercise':ab,ti OR 'resistance aerobic exercise':ab,ti OR 'strength aerobic training':ab,ti OR 'strength endurance training':ab,ti OR 'resistance endurance exercise':ab,ti OR 'concurrent resistance':ab,ti OR 'concurrent strength':ab,ti OR 'concurrent aerobic':ab,ti OR 'concurrent endurance':ab,ti OR 'combined resistance':ab,ti OR 'concurrent training sequence':ab,ti OR 'concurrent training order':ab,ti OR 'combined training order':ab,ti |
| #4 | #1 OR #2 OR #3 |
| #5 | 'fndc5 protein, human':ab,ti OR 'fibronectin type iii domain-containing protein 5 precursor, human':ab,ti OR 'frcp2 protein, human':ab,ti OR 'irisin, human':ab,ti OR fndc5:ab,ti OR irisin:ab,ti |
| #6 | #4 AND #5 |
|  | **中国知网/万方数据库（264）** |
| #1 | 全部：(鸢尾素 OR FNDC5 OR Irisin) |
| #2 | 全部：(同期 OR 同时 OR 结合 OR 联合) |
| #3 | 全部：(有氧 OR 耐力 OR 抗阻 OR 力量 OR 顺序) |
| #4 | #1 AND #2 AND #3 |
