# Supplementary material for: Short- and long-term effects of concurrent aerobic and resistance training on circulating irisin levels in overweight or obese individuals: a systematic review and meta-analysis of randomized controlled trials
Source: PeerJ. 2024 Sep 19;12:e17958. doi: 10.7717/peerj.17958 (PMC11416761; doi:10.7717/peerj.17958)
Supplement: Supplemental Information 2 — CI, confidence interval; SMD, Standardized Mean Difference; CT, concurrent exercise; Con, control; a: Implementing a double-blind randomized controlled trial for exercise interventions is challenging, which may lead to a downgrading of the risk of bias assessment. b: Sample size below optimal information size contributing to imprecision which lowers our certainty in effect. [file peerj-12-17958-s002.docx]

Supplemental Table 2 GRADE evidence profile

| **Certainty assessment** | | | | | | | **Sample** | | **Effect** | **Certainty** | **Importance** |
| --- | --- | --- | --- | --- | --- | --- | --- | --- | --- | --- | --- |
| **Studies** | **Study design** | **Risk of bias** | **Inconsistency** | **Indirectness** | **Imprecision** | **Other considerations** | **CT** | **Con** | **SMD (95% CI)** |  |  |
| **Circulating irisin level** | | | | | | | | | | | |
| 9 | Randomized Controlled Trials | Serious^a^ | Not serious | Not serious | Serious^b^ | none | 153 | 111 | 0.56  (0.33 to 0.80) | ⨁⨁◯◯ Low | CRITICAL |
