# Supplementary figures and images for: Short- and long-term effects of concurrent aerobic and resistance training on circulating irisin levels in overweight or obese individuals: a systematic review and meta-analysis of randomized controlled trials
Source: PeerJ. 2024 Sep 19;12:e17958. doi: 10.7717/peerj.17958 (PMC11416761; doi:10.7717/peerj.17958)

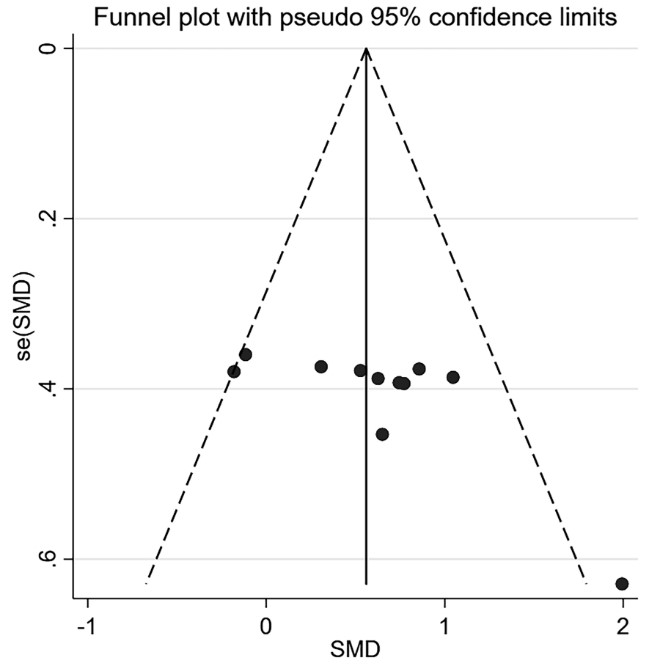

Supplement: Supplemental Information 3 — SMD, Standardized mean difference [file peerj-12-17958-s003.png]

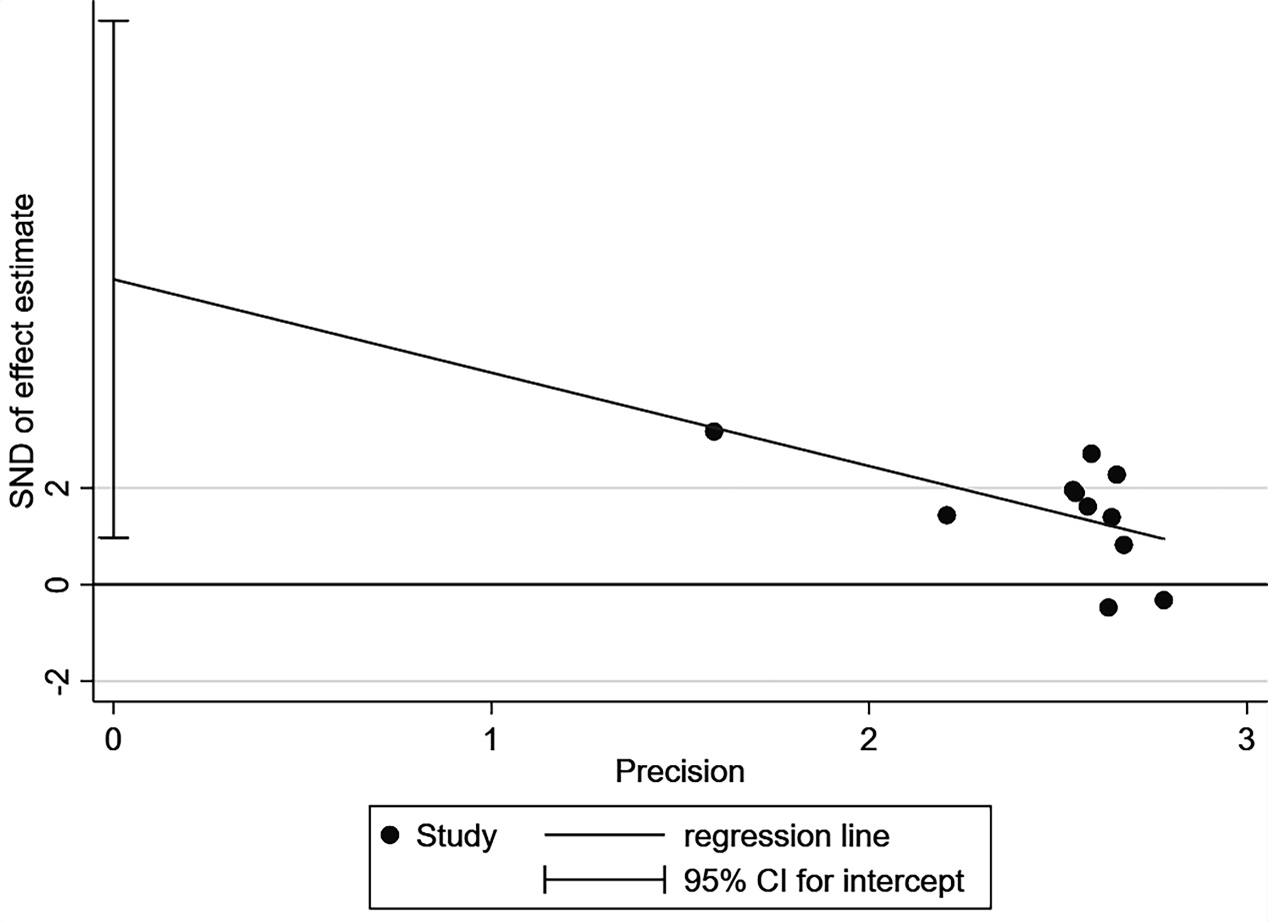

Supplement: Supplemental Information 4 [file peerj-12-17958-s004.png]

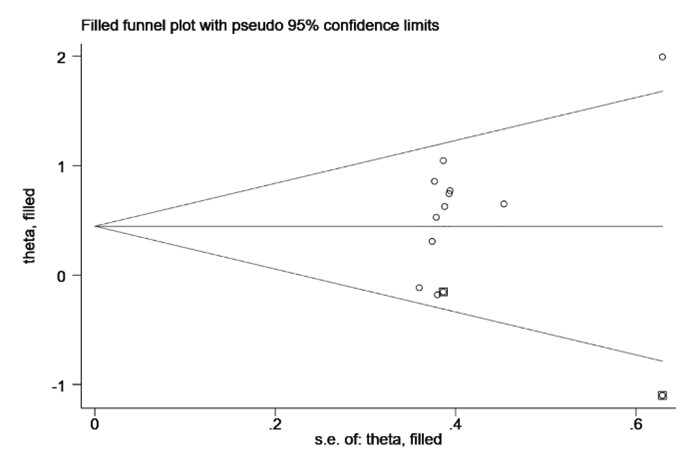

Supplement: Supplemental Information 5 — The adjusted results after accounting for potentially missing studies. [file peerj-12-17958-s005.png]

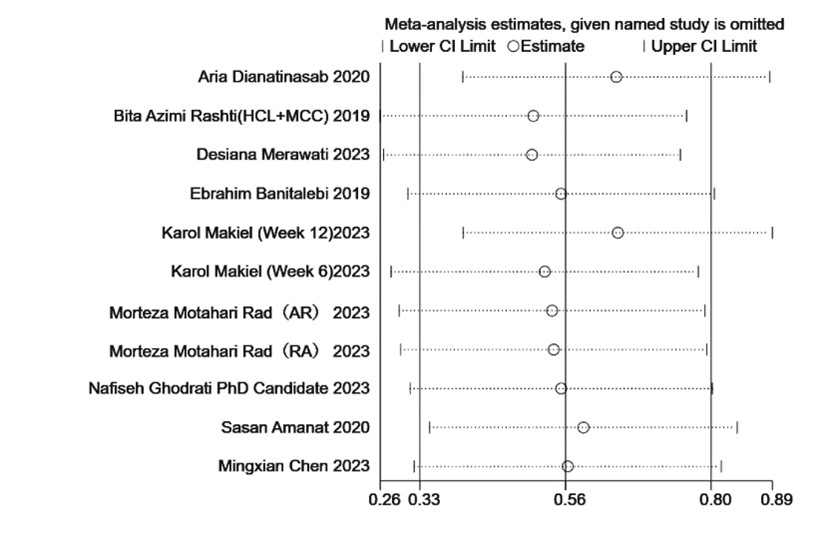

Supplement: Supplemental Information 6 — The reliability of the meta-analysis results remains consistent despite the exclusion of any individual study. Lower Cl Limit, Lower Confidence Limit; Upper Cl Limit, Upper Confidence Limit. [file peerj-12-17958-s006.png]
