# Supplementary material for: Short- and long-term effects of concurrent aerobic and resistance training on circulating irisin levels in overweight or obese individuals: a systematic review and meta-analysis of randomized controlled trials
Source: PeerJ. 2024 Sep 19;12:e17958. doi: 10.7717/peerj.17958 (PMC11416761; doi:10.7717/peerj.17958)
Supplement: Supplemental Information 9 [file peerj-12-17958-s009.docx]

Purpose/Rationale of the systematic review

Irisin is a crucial myokine involved in energy metabolism, with its primary functions including browning of adipose tissue, maintaining glucose homeostasis, and increasing energy expenditure. Given these roles, it holds significant importance for overweight or obese individuals susceptible to chronic diseases. Previous studies have indicated that concurrent training (combining aerobic and resistance training) is an effective method for enhancing both strength and cardiovascular endurance compared to traditional aerobic or endurance training. However, the effects of this training method on irisin are not yet fully understood, particularly in overweight and obese individuals. This study aims to provide insight into the effects of concurrent training on irisin levels, especially in overweight and obese individuals, thereby offering reference to coaches or exercise prescription practitioners in tailoring appropriate exercise regimens to improve the health status of this population.

The contribution that it makes to knowledge in light of previously published related reports, including other meta-analyses and systematic reviews.

Rahimi et al. 's meta-analysis indicates that physical exercise can elevate irisin levels. Additionally, studies have shown that both acute and chronic physical exercise can increase irisin levels (Qiu et al. and Fox et al.). However, concurrent training, as a comprehensive method for improving physical fitness, has been extensively researched. Despite its proven effectiveness in enhancing physical fitness, there is currently neither a systematic evaluation nor a meta-analysis investigating its impact on irisin levels in overweight or obese individuals.
